# Bedside teaching without bedside – an introduction to clinical reasoning in COVID-19 times

**DOI:** 10.3205/zma001410

**Published:** 2021-01-28

**Authors:** Pia Djermester, Christian Gröschke, Robert Gintrowicz, Harm Peters, Antje Degel

**Affiliations:** 1 Charité Universitätsmedizin Berlin, Prodekanat für Studium und Lehre, Berlin, Germany; 2 Vivantes Klinikum Neukölln, Klinik für Innere Medizin, Berlin, Germany; 3 Charité Universitätsmedizin Berlin, Dieter Scheffner Fachzentrum für medizinische Hochschullehre und evidenzbasierte Ausbildungsforschung, Berlin, Germany; 4 Charité Universitätsmedizin Berlin, Med. Klinik für Kardiologie, Campus Benjamin Franklin, Berlin, Germany

**Keywords:** bedside teaching, clinical reasoning, online cases, differential diagnosis

## Abstract

**Introduction: **The Corona virus pandemic rendered most live education this spring term impossible. Many classes were converted into e-learning formats. Teaching at the bedside (BST) seemed unfeasible under the circumstances. BST and clinical reasoning as its major outcome is introduced at the beginning of semester 5, henceforth all BST refers to this first presentation.

**Project outline: **To ensure proficiency of current 5^th^ semester students in future BST sessions, the introduction could not be cancelled albeit teaching with patients was. Knowing that the practical learning objectives of bedside teaching cannot be mirrored in online formats, a compensating module to teach the concept of BST and clinical reasoning had to be designed.

**Summary of work: **To facilitate an understanding of the concept of bedside teaching with a focus on clinical reasoning we developed paper cases and a survey in Microsoft Forms following the history and examination path used in live BST with the addendum of clinical reasoning tables. For the first paper case, a personal feedback was provided for the clinical reasoning tables. A sample solution was provided later for self-feedback on the whole case. The first case was completed by 87, the second by 40 of 336 students. Response to individual feedback was positive. Students still missed hands-on training in history taking and examination with patients.

**Discussion: **Paper cases cannot fully substitute BST. However, given the prime directive during the pandemic to protect our patients, this module engaged around one third of the cohort. The review of uploaded clinical reasoning tables gave proof to the sufficient students’ grasp of clinical reasoning.

**Conclusion: **Albeit not an exhaustive substitute for BST, this online module seems a feasible way to convey clinical reasoning strategies to students.

## Zusammenfassung

**Einführung: **Die Coronavirus-Pandemie machte in diesem Frühjahrssemester den meisten Präsenzunterricht unmöglich. Viele Veranstaltungen wurden in E-Learning-Formate umgewandelt. Der Unterricht am Krankenbett (UaK) schien unter den gegebenen Umständen nicht zulässig. UaK und klinische Entscheidungsfindung als ihr zentrales Lernziel wird zu Beginn von Semester 5 eingeführt, danach bezieht sich jeglicher UaK auf diese erste Einführung.

**Projektskizze:** Um sicherzustellen, dass die derzeitigen Studierenden des 5. Semesters in künftigen UaK-Sitzungen die notwendigen Fähigkeiten beherrschen, konnte die Einführung nicht gestrichen werden, wenngleich Präsenzunterricht mit Patienten nicht durchgeführt wurde. Da die praktischen Lernziele des Unterrichts am Krankenbett nicht in Online-Formaten widergespiegelt werden können, musste ein Ersatzmodul entwickelt werden, um das Konzept des UaK und der klinischen Entscheidungsfindung zu vermitteln.

**Zusammenfassung der Arbeit:** Um das Verständnis des Konzepts des Unterrichts am Krankenbett mit dem Fokus auf der klinischen Entscheidungsfindung zu vermitteln, haben wir Papierfälle und eine Umfrage in Microsoft Forms entwickelt, die dem in Live-UaK verwendeten Anamnese- und Untersuchungspfad mit anhänglichen Tabellen zur klinischen Entscheidungsfindung folgen. Für den ersten Papierfall wurde ein persönliches Feedback zu den Tabellen der klinischen Entscheidungsfindung gegeben. Später wurde eine Musterlösung für das Selbst-Feedback zum gesamten Fall zur Verfügung gestellt. Der erste Fall wurde von 87, der zweite von 40 von 336 Studierenden abgeschlossen. Die Reaktion auf das individuelle Feedback war positiv. Trotzdem vermissten die Studierenden das praktische Üben von Anamnese und Untersuchung mit Patienten.

**Diskussion: **Papierfälle können UaK nicht vollständig ersetzen. Angesichts der während der Pandemie geltenden obersten Direktive, unsere Patienten zu schützen, hat dieses Modul jedoch etwa ein Drittel der Kohorte erfasst. Die Durchsicht der hochgeladenen Tabellen zur klinischen Entscheidungsfindung bewies, dass die Studierenden über ein ausreichendes Verständnis der klinischen Entscheidungsfindung verfügten.

**Schlussfolgerung: **Wenn es auch keinen vollständigen Ersatz für UaK darstellt, so scheint dieses Online-Modul doch ein gangbarer Weg zu sein, den Studierenden Strategien der klinischen Entscheidungsfindung zu vermitteln.

## Introduction

The beginning of spring semester 2020 was postponed due to Corona virus pandemic and teaching thereafter was mostly converted into distance formats [1]. The protection of risk groups by social distancing was decreed by state and federal regulation [2], [3]. This warranted a different approach to Bedside Teaching (BST). This format is at the core of every medical curriculum and an essential step for students to be introduced into their community of practice [4], [5], [6]. 

## Project outline

At Charité - Universitätsmedizin Berlin students are introduced to BST at the beginning of their 5^th^ semester. A preparatory session delineating the process of history taking and physical examination up to the synthesis of main signs into differential diagnoses and further investigations according to clinical reasoning is held in the first two days of the semester. Sophistication and complexity of differential diagnoses (e.g. due to ambiguity of clinical signs) increases over study time until reaching the practical year. Clinical reasoning is an iterative process that needs repetitive case presentations to evolve [7]. This can be achieved via bed-side case-based teaching or paper cases [7], [8]. An important common factor is the feedback and tutorship [9].

## Summary of work

BST as a main format to gain expertise and patterns to recognize was discontinued due to pandemic regulations. Allowing students to work multiple patient cases is crucial for the development of medical expertise [10]. Attempts at online case-based learning have shown promising results in knowledge acquisition and user satisfaction [11]. Thus, we developed two whole case paper cases with unambiguous cardinal presentations (chest pain and dyspnea) as this presentation reduces cognitive load [12], [13] and reflects their current stage in developing expertise, i.e. stage 1 [14]. These included expert evaluation of the presenting problems. In contrast to the cases described by Radon et al. [11] our cases did not contain video or sound files and offered no direct links to background information pertaining to putative diseases. This was mainly due to lack of resources and time. Cases included history and examination information in prose and were to be worked through by the students either individually or in groups according to personal preference. Afterward they were invited to fill out a Microsoft Forms survey in accordance to our patient admission templates. For clinical reasoning, we provided the students with blank tables for differential diagnoses and further investigations ([10], [15], [16]. In the first, differential diagnoses were supposed to be evaluated in accordance to probability on the basis of prior findings. The most probable diagnoses and those most harmful (that should not be missed) were to be identified. In the second table, further investigations had to be identified in accordance to working and most probable differential diseases. Verifying and excluding results were to be anticipated and weighed. Time of case work and completion was left at students´ discretion. Additional tutorials on the concept and its background via video and podcasts were supplied. These podcasts and videos explained the rationale of the concept and specified the intended use of the online forms. Frequently asked questions as well as contact options were provided. Participation in the case surveys was not compulsory.

**Figure 1:**
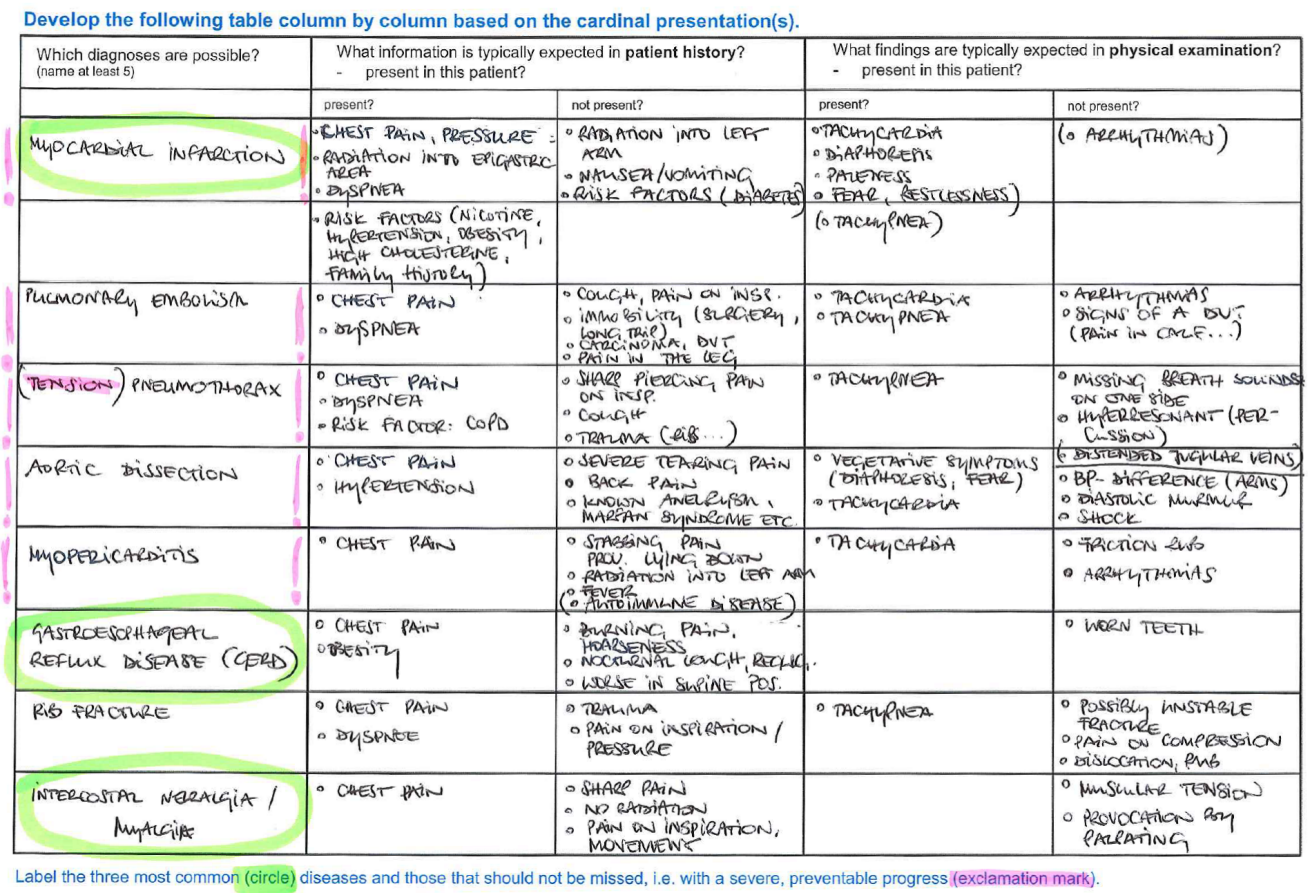
Clinical reasoning – table for the generation of a working and differential diagnoses

**Figure 2:**
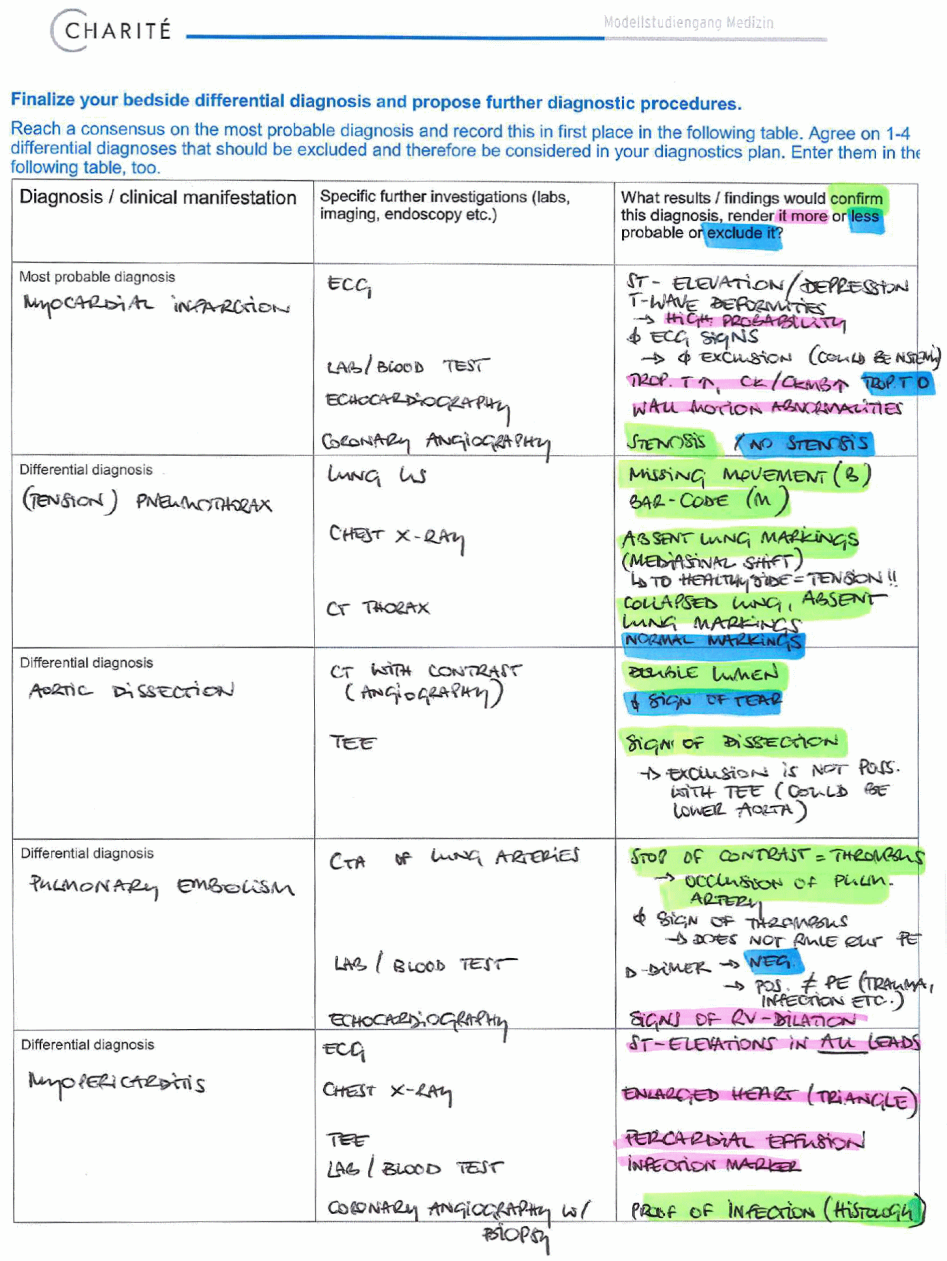
Clinical reasoning – table for the generation of a plan for specific further investigations

The first case was completed by 87 of 336 students, the second by 40 students. Tables uploaded by students (see figure 1 (Fig. 1) and figure 2 (Fig. 2)) were individually reviewed and feedbacked by clinical experts. Most tables showed very complete differential diagnoses tables with clearly recognizable beginnings of reasoning and weighing strategies. Feedback concerned the uploaded differential diagnoses and diagnostics tables, this was supplied via email. The feedback included a general part again emphasizing again the importance of clinical reasoning and – in absence of pattern recognition due to lack of expertise – system 2 reasoning. An individual comment on the completion of the tables as well as important red flags that were missed and learning gaps completed the feedback. This was appreciated by those students concerned as mentioned in short evaluations via email replies (e.g. “helps prioritizing in future cases”, “had not expected such a constructive feedback” and “will try to follow your lead […] using the templates and labels to gain a better weighting and overview”). Still hands-on training in history taking and physical examination with real patients was sorely missed.

## Discussion

An online tutorial and paper cases with self-study and clinical reasoning tables to facilitate the initial analytical process and support transition to dual process reasoning [17] is an imperfect alternative to BST. It may convey basic clinical reasoning strategies – but it lacks many components that make BST a rich and valuable format, e.g. hands-on skill training in history taking and physical examination, communication as well as role modeling for desirable attitudes [6], [18], [19], [20], [21], [22], [23]. It represents the students’ initiation into their future community of practice. It also seems that patients value their sessions of BST [23], [24]. 

An important advantage of the online module is the homogeneity of administration and feedback since all students received the same case and those that uploaded answers were feedbacked by a small group of clinical teachers. In a vast and heterogenous faculty with a significant employee turnover the chance of a consistent and standardized initiation into clinical reasoning has proven difficult as displayed in faculty development programs. Adding multimodal resources such as video or sound files to the cases could further decrease extraneous cognitive load and add authenticity to the cases [12]. Online cases can cover a multiplicity of cases including increasing complexity and ambiguity as well as adjacent diseases thus enabling illness script generation and reflective reasoning [14], [25]. Combining these online cases with (online) standardized patient interaction and/or elaboration with a clinical teacher in a small group (also online) could further aid in gaining knowledge structures needed for expertise development [10].

The reason for dwindling of participation for case 2 is not clear but could be related to a latter publication which may have collided with a lessening of restrictions and preparation for exams. 

Our online module seems to be apt to support clinical reasoning training possibly as a blended format but cannot fully replace BST under normal circumstances. We hope that with this training our students can progress into advanced BST modules mastering basic clinical reasoning. The clinical reasoning online cases will be further developed to include multimodal sources and cover important presentations and diseases. 

Further research into the effectiveness in conveying clinical reasoning skills needs to be conducted possibly in comparison with the historical cohort lacking the online instruction.

## Conclusions

Paper cases with tables on differential diagnoses in the sense of probability matrices may help facilitate clinical reasoning in the absence of BST as warranted by COVID-19 decrees and can constitute a valuable add-on to standardize clinical reasoning learning blended with regular BST under normal circumstances.

## Competing interests

The authors declare that they have no competing interests. 

## References

[1] Der Regierende Bürgermeister - Senatskanzlei Wissenschaft und Forschung (2020). Aktuelle Informationen zu Corona-Maßnahmen an Hochschulen und Forschungseinrichtungen - Berlin.de.

[2] Bundesministerium für Gesundheit (2020). Coronavirus SARS-CoV-2: Chronik der bisherigen Maßnahmen.

[3] Der Regierende Bürgermeister - Senatskanzlei (2020). Eindämmung des Coronavirus - Berliner Senat beschließt weitgehende Kontaktbeschränkungen.

[4] Ramani S (2003). Twelve tips to improve bedside teaching. Med Teach.

[5] Ramani S, Orlander J, Strunin L, Barber T (2003). Whither Bedside Teaching? A Focus-group Study of Clinical Teachers. Acad Med.

[6] Ramani S, Orlander JD (2013). Human dimensions in bedside teaching: focus group discussions of teachers and learners. Teach Learn Med.

[7] Kassirer JP (2010). Teaching Clinical Reasoning: Case-Based and Coached. Acad Med.

[8] Irby DM (1994). Three exemplary models of case-based teaching. Acad Med.

[9] Thistlethwaite JE, Davies D, Ekeocha S, Kidd JM, MacDougall C, Matthews P, Purkis J, Clay D (2012). The effectiveness of case-based learning in health professional education. A BEME systematic review: BEME Guide No. 23. Med Teach.

[10] Schmidt HG, Rikers RM (2007). How expertise develops in medicine: knowledge encapsulation and illness script formation. Med Educ.

[11] Radon K, Kolb S, Reichert J, Baumeister T, Fuchs R, Hege I, Praml G, Fischer M, Nowak D (2006). Case-based e-learning in occupational medicine--The NetWoRM Project in Germany. Ann Agric Environ Med.

[12] Van Merriënboer JJ, Sweller J (2010). Cognitive load theory in health professional education: design principles and strategies. Med Educ.

[13] Nendaz MR, Raetzo MA, Junod AF, Vu NV (2000). Teaching Diagnostic Skills: Clinical Vignettes or Chief Complaints?. Adv Health Sci Educ Theory Pract.

[14] Schmidt HG, Mamede S (2015). How to improve the teaching of clinical reasoning: a narrative review and a proposal. Med Educ.

[15] Schmidt HG, Boshuizen HP (1993). On the origin of intermediate effects in clinical case recall. Mem Cognit.

[16] Verkoeijen PP, Rikers RM, Schmidt HG, Van De Wiel MW, Kooman JP (2004). Case representation by medical experts, intermediates and novices for laboratory data presented with or without a clinical context. Med Educ.

[17] Norman G (2009). Dual processing and diagnostic errors. Adv Health Sci Educ Theory Pract.

[18] Alpert JS (2009). Some thoughts on bedside teaching. Am J Med.

[19] Chapman R, Wynter L, Burgess A, Mellis C (2014). Can we improve the delivery of bedside teaching?. Clin Teach.

[20] Chretien KC, Goldman EF, Craven KE, Faselis CJ (2010). A Qualitative Study of the Meaning of Physical Examination Teaching for Patients. J Gen Int Med.

[21] Jones P, Rai BP (2015). The status of bedside teaching in the United Kingdom: the student perspective. Adv Med Educ Pract.

[22] Mookherjee S, Chou CL (2011). Bedside teaching of clinical reasoning and evidence-based physical examination. Med Educ.

[23] Nair BR, Coughlan JL, Hensley MJ (1997). Student and patient perspectives on bedside teaching. Med Educ.

[24] Monrouxe LV, Rees CE, Bradley P (2009). The construction of patients' involvement in hospital bedside teaching encounters. Qual Health Res.

[25] Mamede S, Schmidt HG, Rikers RM, Penaforte JC, Coelho-Filho JM (2007). Breaking down automaticity: case ambiguity and the shift to reflective approaches in clinical reasoning. Med Educ.

